# Examination of game addiction studies conducted in Turkey: A systematic review study

**DOI:** 10.3389/fpsyt.2023.1014621

**Published:** 2023-04-14

**Authors:** Canahmet Boz, Mehmet Dinç

**Affiliations:** Department of Psychology, Faculty of Economics, Administrative and Social Sciences, Hasan Kalyoncu University, Gaziantep, Türkiye

**Keywords:** game addiction, digital game addiction, internet game addiction, online game addiction, systematic review

## Abstract

The aim of this study is to examine the methodological orientations in game addiction studies in Turkey between 2019 and 2022 in a holistic way and to present suggestions for new literature studies. For this purpose, only articles written in Turkish language were reviewed on Google Scholar. Open-access quantitative studies between 2019 and 2022 were included in our study. As a result, 69 out of 257 studies were found to comply with the predetermined criteria. The number of participants in these 69 studies, with a total sample size of 26,415, varies between 60 and 987. Almost half of the studies sample group is children (*n* = 36). The majority of the studies examine the relationship between academic achievement, familial and social relationship problems, gaming behavior and game addiction in children and adolescents. It was found that gaming addiction was higher in male students compared to female students, especially between the ages of 14–15. Among children, male students play digital games more than girls, and they mostly prefer action-adventure, war, and racing games. Previous studies have concluded that the use of consoles and smartphones in adolescents and young adults has a high impact on digital game addiction. In the light of the findings, it is suggested that empirical studies on game addiction should be addressed with different aspects using new literature studies.

## Introduction

While technological devices and the internet are used as a facilitating tool in many aspects of life, their use for games and entertainment has become quite widespread. It is found that digital games, which have users in almost every segment of the society, are widespread especially among young people (1). In this respect, some individuals may lose control over gaming to the extent that it becomes problematic (2). In the 11th revision of the International Disease Classification (ICD-11), gaming disorder is defined as a gaming behavior model characterized by loss of control over gaming (3). According to the diagnosis, it has been stated that the occurrence of 5 of the following 9 items in the last 12 months can be mainly evaluated in the diagnosis. These criteria are: (1) Constantly dealing with internet games mentally. (2) The emergence of withdrawal symptoms when internet games are not accessible. (3) Willingness to spend longer periods gaming and increased tolerance. (4) Unsuccessful attempts to control the gaming behavior. (5) Loss of interest in old hobbies and entertainment due to playing internet games. (6) To continue the games excessively despite the problems that arise. (7) Lying to his/her family, therapist and environment about the duration of playing internet games. (8) Playing internet games to escape or get rid of negative emotional states (such as guilt, anxiety). (9) To jeopardize or give up an important relationship, education or job opportunity to play internet games.

When we take look at the prevalence of playing games around the world, on average, 51% of Europeans play video games, whereas 59% of them use smartphones or tablets while playing video games (4). The rate of gaming disorder in Europe is reported to be between 1 and 10% (5–8). In addition, in a study conducted among young people in East and Southeast Asian countries, the prevalence of gaming disorder was found to be around 10–15% (9). In a study conducted in Switzerland in 2015, 15% of individuals between the ages of 15–34 (10); 14% of adults in Republic of Korea (11) and 17% of participants in a study conducted with secondary school students in Iran (12) were found to develop gaming disorders.

According to the Gaming in Turkey Game/E-Sports Agency’s Game Sector in Turkey 2020 Report, 79% of adults in Turkey play digital games (13). According to a comprehensive study conducted throughout Turkey in 2019, the rate of individuals with a high risk of digital game addiction was found to be 32%. In addition, it has been stated that individuals between the ages of 18–23 living in Eastern and Southeastern Anatolia regions have a higher risk (14). Considering the data, an increased risk of game addiction is seen. In this vein, the importance of the study is that gives direction to new research by examining the previous literature on game addiction in Turkey.

In this study, it was aimed to examine the variables, methodological trends and analysis methods of digital game addiction studies conducted in Turkey between 2019 and 2022 (July 2022). In this context, the questions of our study are determined as follows.

1. What are the methodologies used in digital game addiction studies?
2. What are the characteristics of the samples in digital game addiction studies?
3. What are the data analysis methods in digital game addiction studies?
4. What are the variables in digital game addiction studies and what are the variables that are effective and are not effective?

## Method

### Search strategies

The study data (“Game Addiction” and “GA”), (“Digital Game Addiction” and “DGA”), (“Internet Game Addiction” and “IGA”), (“Computer Game Addiction” and “CGA”) terms were only reviewed in the literature by marking the “Search in Turkish pages” option on Google Scholar. The study data were collected only from the research data written in Turkish language. For this reason, no search was made through the Web of Science database. Studies published between 2019 and July 2022 were examined. Previously, there was a systematic review study on digital game addictions by Şimşek and Yılmaz (15) between 2010 and 2018. For this reason, it was decided that the date range of our study will cover the period between 2019 and 2022.

### Study selection

**Figure 1:**
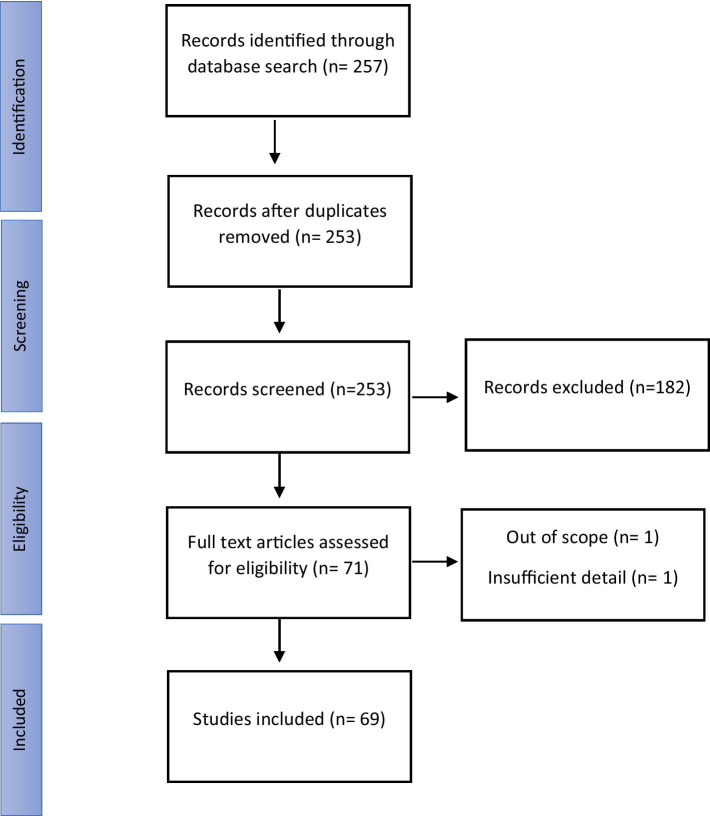
Diagram showing the information flow through the review: the number of records identified, included, and excluded.

After the literature review, a total of 257 open-access articles, papers and thesis studies were found. The thesis and paper studies were not included in the study because they did not undergo a double-blind review process. After the procedure, a total of 69 articles were found to comply with the following criteria (Figure 1):

1. Published between 2019 and July 2022,
2. A quantitative research article,
3. Published in Turkish language (It is aimed to contribute to the international literature), and
4. Open access.

### Data analysis

In systematic analysis, basic information about the study is analyzed in accordance with the purpose of the study (16). All the studies collected were numbered and listed as publication title, sampling type, sample group, sample size, study variables and analysis type with a table on Microsoft Excel. After the listing, the studies to be included were determined by considering the predetermined criteria. After the analysis procedures were completed, the findings were presented according to the methodological trends in digital game addiction studies and research questions related to digital game addiction.

## Results

### Methodological trends in digital game addiction studies

As seen in Table 1, the titles of 69 publications, sample group, sample size and study variables are specified.

**Table 1:** Methodological trends in digital game addiction studies.

Number	Reference	Sample group	Sample size	Variables
1	(17)	Adolescents (High school students)	271 (M:120 F:151)	*Smartphone *Game Addiction
2	(18)	Children (Secondary school)	279 (M: 136 F:143)	*Digital Gaming Addiction *Aggression
3	(19)	Children (Primary and secondary school)	321 (M: 173 F:148)	*Computer Game Addiction *Alexithymia *Social Anxiety
4	(20)	Children (Secondary school)	240 (M: 115 F:125)	*Digital Game Addiction *Peer Bullying Cognitions *Empathy
5	(21)	Children (Secondary school)	331 (M: 180 F: 151)	*Computer Game Addiction *Empathy *Alexithymia
6	(22)	Adolescents and young adults (14–20 years old athletes)	714 (M: 536 F:178)	*Computer Game Addiction *Psychological Skills
7	(23)	Children (Secondary school)	600 (M: 287 F:313)	*Digital Game Addiction * Subjective Well-Being in School
8	(24)	Adolescents (16–18 years)	60	*Digital Game Addiction *Maternal Attitude * Self-Control
9	(25)	Young adults (University students)	113 (M: 29 F: 84)	*Digital Game Addiction *Healthy Life Parameters
10	(26)	Adolescents (12–18 years)	658 (M: 335 F: 323)	*Digital Game Addiction * Browsing the Internet for No Purpose
11	(27)	Adolescents (12–18 years)	400 (M: 158 F: 242)	*Online Gaming Addiction *Peer Bullying
12	(28)	Young adults (University students)	396 (M: 301 F: 95)	*Online Gaming Addiction *Aggression
13	(29)	Children (Secondary school)	435 (M: 233 F:202)	*Digital Game Addiction *Self-efficacy belief levels
14	(30)	Children (Secondary school)	276 (M: 117, F:159)	*Gaming Addiction *Gaming duration and type
15	(31)	Young adults (E-sport players and athletes)	141 (M: 105 F:36)	*Game Addiction *Digital Game Addiction Awareness
16	(32)	Adolescents and young adults (14, 15, 16, 17, 18, 19 years old)	479 (M: 240 F: 239)	*Gaming Addiction *Frequency of Internet use *Violent games * Education level of parents
17	(33)	Children (10–14 years)	352 (M: 183 F: 169)	*Digital Game Addiction *Game type
18	(34)	Children (Secondary school)	352 (M:154 F: 198)	*Computer Game Addiction *Grade level *Family income level * Having a personal computer
19	(35)	Children (Primary school)	154 (M: 73 F: 81)	*Computer Game Addiction *Behavioral Problems * Competence in school and out-of-school activities
20	(36)	Children (Secondary school)	168 (M: 88 F:80)	*Digital Gaming Addiction *Self-control skills
21	(37)	Children (Secondary school)	409 (M: 217 F: 192)	*Digital Gaming addiction *Bullying
22	(38)	Children (Secondary school)	224 (M: 109 F:115)	*Digital Gaming Addiction *Self-Perception *Grade level, Having a computer *Number of siblings *Parental education level
23	(39)	Children, adolescents, adults (12–61 years old)	726 (M: 679 F: 47)	*Online Gaming Addiction *Multidimensional social support *Life satisfaction *Self-esteem
24	(40)	Children (8–13 years)	516 (M: 283 F:233)	*Computer Game Addiction *Levels of Tendency to Violence
25	(41)	Adolescents (High school students)	446 (M: 240 F:206)	*Digital Gaming Addiction *Level of Happiness
26	(42)	Children (Secondary school)	286 (M: 150 F:136)	*Tendency to Play Digital Games *Computer Game Addiction Level
27	(43)	Young adults (University students)	453 (M: 339 F:114)	*Computer Game Addiction *Sociodemographic Data
28	(44)	Children (Secondary school)	333 (M: 140 F: 193)	*Digital Gaming Addiction *Sociodemographic Data
29	(45)	Young adults (University students)	509 (M: 290 F:219)	*Digital Gaming Addiction *Sociodemographic Data
30	(46)	Children (Primary school)	230 (M: 109 F:121)	*Digital Gaming Addiction *Sleep
31	(47)	Adolescent (High school students)	303 (M: 244 F: 59)	*Digital Gaming Addiction *Smartphone Addiction *Computer Addiction
32	(48)	Children (Secondary school)	945	*Computer Game Addiction * Protective Factors in the Family
33	(49)	Children (Secondary school)	635 (M: 319 F: 316)	*Computer Game Addiction *Parental Behaviors
34	(50)	Young adults, adults (20–40 years)	497 (M: 279 F: 218)	*Digital Gaming Addiction *Sociodemographic Data
35	Kestane and İlgar (51)	Children (Primary SCHOOL)	697 (M: 329 F:368)	*Digital Gaming Addiction *Sociodemographic Data
36	Öztürk and Dalaman (52)	Children (Primary school)	137 (M: 66 F:71)	*Computer Game Addiction *Sociodemographic Data
37	(53)	Children (6–12 years)	69 (M: 42 F: 27)	*Computer Game Addiction *Sociodemographic Data
38	(54)	Children (10–11 years)	395 (M:201 F:194)	*Digital Addictions *Coordinative and Conditional Capabilities
39	(55)	Adolescents	583 (M: 278 F:305)	*Gaming Addiction *Physical activity attitudes and behaviors
40	(56)	Children (Primary school) and their parents	123 (M: 59 F:64)	*Gaming Addiction *Sleep *Academic Achievement
41	(57)	Adolescents (High school students)	160	*Gaming Addiction *Psycho-education program based on Motivational Interviewing Technique
42	(58)	Children (Secondary school)	650 (M: 330 F:320)	*Digital Gaming addiction *Bullying Cognitions
43	(59)	Young adults (University Students)	517 (M: 334 F: 183)	*Digital Gaming Addiction *Sociodemographic Data
44	(60)	Children (9–11 years)	300 (M: 129 F:171)	*Computer Game Addiction *Behavioral Problems
45	(61)	Children (9–14 years)	100 (M: 44 F:56)	*Digital Gaming Addiction *Physical Activity *Sleep habits
46	(62)	Adolescents (14–18 years)	134 (M: 50 F:84)	*Gaming Addiction *Character Development
47	(63)	Adolescents (13–18 years)	162 (M: 59 F:103)	Happiness and meaning of life
48	(64)	Children (Secondary school)	987 (M: 467 F:520)	*Digital Gaming Addiction *Game type *Gaming duration
49	(65)	Young adults (University students)	254 (M: 197 F:57)	*Digital Gaming Addiction *Sociodemographic Data
50	(66)	Young adults (University students)	234 (M: 90 F:144)	*Online Gaming Addiction *Stress *Anxiety *Depression *Academic Achievement
51	(67)	Children (Secondary school)	286 (M:161 F:125)	*Virtual Gaming Addiction *Sociodemographic Data
52	(68)	Adolescents (12–18 years)	639 (M: 345 F:294)	*Online Gaming Addiction *Perceived Stress *Perceived Social Support
53	(69)	Young adults (University students)	160 (M: 62 F:98)	*Digital Gaming Addiction *Sociodemographic Data
54	(70)	Children and adolescents (Primary and secondary schools)	939 (M: 417 F:522)	*Digital Gaming Addiction *Sociodemographic Data
55	(71)	Children and adolescents (Secondary and high school)	500 (M: 292 F:208)	*Digital Gaming Addiction *Sociodemographic Data
56	(72)	Adolescents (14–18 years)	385 (M:175 F:210)	*Digital Gaming Addiction *Family Life Satisfaction
57	(73)	Adolescents (15–17 years)	300 (M:190 F:110)	*Internet Game Addiction *Perceived Social Support *Gaming Duration *Dysfunctional Belief
58	(74)	Adolescents (High school students)	312 (M:187 F:125)	*Digital Gaming Addiction *Sleep
59	(75)	Adults (parents)	423	*Digital Game Addiction *Parental Attitudes
60	(76)	Adolescent (High school students)	613 (M:212 F:401)	*Digital Game Addiction *Loneliness Levels
61	(77)	Young adults (University students)	398 (M: 121 F:277)	*Gaming Addiction *Personality Traits *Stress Coping Style
62	(78)	Children and adolescents (Secondary and high school)	389 (M: 389 F:0)	*Digital Game Addiction * Negative social skills in children *Social behaviors
63	(79)	Children (12–15 years)	248 (M: 124 F:124)	*Gaming Addiction *Smartphone Addiction *Social Media Addiction
64	(80)	Children and adolescents (Secondary and high school)	478 (M: 191 F:287)	*Digital Game Addiction *Religiosity
65	(81)	Children and adolescents (Secondary and high school)	866 (M: 435 F:431)	*Digital game addiction *Parental attitude
66	(82)	Children (Primary school)	109 (M: 54 F:55)	*Computer Game Addiction *Chronotype Sleep
67	(83)	Adolescent (High school students)	258 (M: 119 F:139)	*Digital Game Addiction *Leisure boredom
68	(84)	Young adults (University students)	248 (M:139 F:109)	*Digital Game Addiction
69	(85)	Young adults (University students)	110 (M: 54 F: 56)	*Internet Gaming Addiction *Aggression *Coping Strategies

As a result of the literature review, there are 69 studies that meet the criteria. The total sample size of 69 studies was 26,415. Many different types of samples were used, and the number of participants in the studies varied between 60 and 987. Most of the studies have studied large sample groups. The sample group of almost half of the studies consisted of children (*n* = 36). The remaining sample groups consisted of studies with adolescents or those involving adolescents (*n* = 21) and studies with young adults or those involving young adults (*n* = 15).

Data collection tools used to measure game addiction levels are presented in Table 2. In these studies, valid and reliable scales were utilized. Especially since 2020, new scales have been developed and started to be widely used with the concept of game addiction finding more place in the literature. The Computer Game Addiction Scale for Children and the Digital Gaming Addiction Scale for Children are among the commonly used scales. In the 16 study “Computer Game Addiction Scale for Children,” in 16 study “Digital Game Addiction Scale for Children,” in the 14 study “Digital Game Addiction Scale,” in the 9 study “Game Addiction Scale for Adolescents,” in the 5 study “Online Gaming Addiction Scale,” in the 4 study “Digital Gaming Addiction Scale for University Students,” in the 2 study “Internet Gaming Disorder Scale Short Form” and in the 2 study “Gaming Addiction Scale” was used.

**Table 2:** Scales used in relation to game addiction.

Name of the scale	Scale developer-adaptor	*N*
Computer game addiction scale for children	(86)	16
Digital game addiction scale for children	(87)	16
Digital game addiction scale	(88)	14
Game addiction scale for adolescents	(89)	9
Online gaming addiction scale	Kaya (90)	5
Digital gaming addiction scale for university students	(87)	4
Internet gaming disorder scale short form	(91)	2
Gaming addiction scale	(92)	2

Children and adolescents have a higher risk of gaming addiction. However, it was found that gaming addiction was higher in male students compared to female students, especially between the ages of 14–15 (in the 24 study). It was concluded that the levels of digital game addiction and aggression were high in children whose gaming duration was not restricted by parents. Among children, male students play digital games more than girls, and they mostly prefer action-adventure, war, and racing games. As children’s digital game addiction increases, their subjective well-being, and self-efficacy levels tend to decrease. Studies conducted with children have demonstrated that social anxiety significantly affects computer game addiction in children. In addition, it can be purported that computer game addiction and the tendency to violence in children may increase with the prolonged playing of violent computer games. Similarly, studies conducted with children have shown that most of the children play games from mobile devices; most students play games at home; they mostly prefer educational games, and they usually play games while traveling and in their spare time.

It was observed that adolescents who played excessive digital games had lower levels of self-control. In addition, it has been revealed that adolescents watching violent movies have higher levels of game addiction. Moreover, previous studies have concluded that the use of consoles and smartphones in adolescents and young adults has a high impact on digital game addiction.

It has also been observed that anxiety can lead to digital game addiction in young adults and those with aggressive behaviors have a higher risk of game addiction. When the duration of playing digital games before and during the pandemic in young adults was compared, it was found that this duration increased significantly during the pandemic, that digital games were played mostly by phone, and that the pandemic process increased the rate of playing war strategy games. According to a study conducted with young adults, it was found that males had a higher risk of being addicted to digital games than females, that 58% of university students chose mobile platforms to play digital games, that the most preferred game genre was First-Person Shooter (FPS) genre (31%), and that 80.7% of the students participated in the study had low-risk and risky scores in terms of addiction.

## Discussion

In this study, the studies conducted in the literature were reviewed in order to present new ideas and draw a road map for the studies on game addiction in Turkey. When the studies on game addictions are examined, it is seen that the studies conducted in certain areas make the literature saturated.

According to previous studies, digital game addiction has been shown to have a negative effect on physical and psychological health, well-being, functionality, work, school and private life (93–95). When the literature was examined, it was seen that gaming addiction had negative effects on children and adolescents’ academic achievement, family life and social relations in parallel with our systematic review (96–98). On the other hand, as in children and adolescents, there are negative effects of game addiction in adults (99–101). Considering the studies conducted with adults, it is noteworthy that there are few studies conducted with adults in Turkey. The majority of the studies studied children and adolescents. The effects of game addiction on adults need to be further examined in Turkey.

Considering the studies conducted, it is of great importance to select the correct and appropriate sample although the selected sample sizes are sufficient (102). In the majority of the studies, it was observed that more easily accessible sample groups were selected. As the sample size increases, it is seen that the statistical significance increases especially in correlational studies. For this reason, it is important that the studies designed have randomization and control groups in order to obtain more qualified results. When the studies were examined, it was seen that they recruited the participants with random sampling method. In addition, these studies should be supported by regression studies, beyond correlation studies. In order to obtain more accurate and generalizable data, experimental studies with control groups and random assignment should be designed.

When the variables and results discussed in these studies were examined, it was seen that correlational analyzes were used for variables such as gender, age, parental education status, having a computer, grade level, academic achievement, and the ages of the participants. These variables are also widely discussed in the global literature. When the studies were examined, it was determined that males were more addicted to digital games or riskier than females in terms of gender. Literature shows that boys are more interested in computer games than girls, that the level of game addiction was higher than girls, and that they were more exposed to the effects of computer games—all of which are in line with the findings of the current research (103–105). Similarly, considering the previous studies, it was observed that children and adolescents who perceived that they had social support had a lower risk of game addiction. When the global literature is examined, it is seen that social support reduces game addiction levels as it positively affects psychosocial well-being (106, 107).

As a result, this study has made suggestions on the elements to be considered in the digital game addiction studies conducted in Turkey. During the assessment process of this study, new studies were conducted in accordance with some of the suggestions in the discussions of this study. Especially in children and adolescents, there are studies examining the relationship between academic achievement, familial and social relationship problems, gaming behavior and game addiction. However, there is still a need for studies that are supported by quantitative methods that will provide an in-depth understanding of addiction, and whose methodologies are clear and appropriate. Finally, it can be suggested to future empirical studies to address different dimensions of digital game addiction with new literature studies.

### Limitation of study

This study has various limitations. Firstly, the study is limited to the studies conducted in Turkey between certain years and reviewed in certain databases. In the preparation phase of the study, studies conducted until 2022 were addressed. Finally, only quantitative data were included in the study.

## Data availability statement

The original contributions presented in the study are included in the article/supplementary material, further inquiries can be directed to the corresponding author.

## Author contributions

MD drafted the initial version of the manuscript and provided the comments. CB collected the data and analyzed the data. All authors contributed to the article and approved the submitted version.

## Conflict of interest

The authors declare that the research was conducted in the absence of any commercial or financial relationships that could be construed as a potential conflict of interest.

## Publisher’s note

All claims expressed in this article are solely those of the authors and do not necessarily represent those of their affiliated organizations, or those of the publisher, the editors and the reviewers. Any product that may be evaluated in this article, or claim that may be made by its manufacturer, is not guaranteed or endorsed by the publisher.
